# Hearing Impairment and Neuroimaging Results in Mitochondrial Diseases

**DOI:** 10.3390/jpm13091329

**Published:** 2023-08-29

**Authors:** Gabriella Cadoni, Guido Primiano, Pasqualina M. Picciotti, Rosalinda Calandrelli, Jacopo Galli, Serenella Servidei, Guido Conti

**Affiliations:** 1 Dipartimento di Neuroscienze, Organi di Senso e Torace, Fondazione Policlinico Universitario Agostino Gemelli IRCCS, 00168 Rome, Italy; gabriella.cadoni@unicatt.it (G.C.); guido.primiano@policlinicogemelli.it (G.P.); jacopo.galli@unicatt.it (J.G.); serenella.servidei@unicatt.it (S.S.); guido.conti@unicatt.it (G.C.); 2 Dipartimento di Testa-Collo e Organi di Senso, Catholic University of the Sacred Heart, 00168 Rome, Italy; 3 Dipartimento di Neuroscienze, Università Cattolica del Sacro Cuore, 00168 Rome, Italy; 4 Dipartimento di Diagnostica per Immagini, Radioterapia Oncologica ed Ematologia, Fondazione Policlinico Universitario Agostino Gemelli IRCCS, Università Cattolica del Sacro Cuore, 00168 Rome, Italy; rosalinda.calandrelli@policlinicogemelli.it

**Keywords:** mitochondrial diseases, mtDNA, deafness, cochlear, retrocochlear, MRI, brain changes

## Abstract

Mitochondrial diseases (MDs) are heterogeneous genetic disorders characterized by mitochondrial DNA (mtDNA) defects, involving tissues highly dependent on oxidative metabolism: the inner ear, brain, eye, skeletal muscle, and heart. We describe adult patients with genetically defined MDs, characterizing hearing function and neuroimaging results. We enrolled 34 patients (mean age: 50.02 ± 15 years, range: 18–75 years; 20 females and 14 males) classified in four groups: MELAS, MIDD, PEO, and Encephalopathy/Polyneuropathy. Audiological evaluations included psychoacoustical tests (pure-tone and speech audiometry), electrophysiological tests (Auditory Brainstem Responses, ABRs), and Impedenzometry. Neuroimaging evaluations considered global MRI abnormalities or structural brain changes. In total, 19/34 patients carried the m.3243A > G mutation (6 affected by MELAS, 12 affected by MIDD, and 1 affected by PEO); 11 had an mtDNA deletion (all affected by PEO); 3 had nuclear genes associated with MDs (POLG1 and OPA1); and 1 patient had an mtDNA deletion without an identified nuclear gene defect (affected by PEO). Sensory neural, bilateral, and symmetrical hearing loss was present in 25 patients (73.5%) to different degrees: 9 mild, 9 moderate, 5 severe, and 2 profound. The severe/profound and mild hearing losses were associated with pantonal and high-frequency audiograms, respectively. Instead, moderate hearing losses were associated with both high-frequency (five cases) and pantonal (five cases) audiogram shapes. In addition, 21/25 patients showed a cochlear site of lesion (84%), and 4/25 (16%) showed a retrocochlear site. We found global MRI abnormalities or structural brain changes in 26/30 subjects (86.6%): 21 had white matter abnormalities, 15 had cortical atrophy, 10 had subcortical atrophy, 8 had basal nuclei involvement or cerebellar atrophy, 4 had stroke-like lesions or laminar necrosis, and 1 had cysts or vacuolated lesions. We concluded that genetic alterations are associated with different clinical presentations for both auditory function and neuroradiological findings. There is no fixed relationship between genotype and phenotype for the clinical conditions analyzed.

## 1. Introduction

In recent years, the knowledge of mitochondrial diseases (MDs) has greatly increased. MDs are complex disorders associated with an abnormal cellular respiratory chain and oxidative phosphorylation for adenosine triphosphate (ATP) synthesis.

Their phenotypes can be different, ranging from a myopathy to multisystem diseases. The age of onset, severity, and clinical progression can also be highly variable.

On the other hand, their genetic features can also vary. Genetic defects can be located in the mitochondrial DNA (mtDNA) or the nuclear genome (nDNA). Mitochondrial disorders due to mtDNA mutations follow a different inheritance than nuclear genes. Single mtDNA deletions can be sporadic, while other mtDNA point mutations can be inherited according to the rules of mitochondrial genetics (maternal inheritance, mitotic segregation, heteroplasmy, and threshold effect) [1].

MDs are the most common inherited neurometabolic disorders. Pathogenic variants of nuclear or mtDNA variants are estimated to affect 1 in 4300 individuals [1]. Patients can be diagnosed and managed by different specialists.

In addition to the considerable phenotypic and genotypic variability, there is an important and variable genotype–phenotype relationship (a single mutation can cause several clinical syndromes, while different genetic alterations can cause similar phenotypes). This makes MD evaluation very complex and justifies the large number of syndromic presentations.

Mitochondria are ubiquitous, which explains large variability typical of MDs showing different clinical characteristics. Tissues that are highly dependent on oxidative metabolism are most frequently involved, and the effects of mutations affecting the respiratory chain are often multisystemic. The involvement of the visual and auditory pathways, heart, central nervous system, and/or skeletal muscle is well known. Therefore, a mitochondrial disorder should be considered in the presence of myopathy with exercise intolerance, axonal neuropathy, eyelid ptosis, ophthalmoparesis, pigmentary retinopathy, optic neuropathy, sensorineural hearing loss, diabetes mellitus, hypertrophic cardiomyopathy, migraine, short stature, and cognitive impairment [1].

In MDs, hearing impairment may be non-syndromic, as deafness is an isolated symptom, or associated with other disorders in syndromic hearing loss. Syndromic hearing loss includes Mitochondrial Encephalomyopathy, lactic acidosis, and stroke-like episodes (MELAS); maternally inherited diabetes with deafness (MIDD); chronic progressive external ophthalmoplegia (CPEO); and Myoclonic Epilepsy with Ragged-Red Fibers (MERRF) [2,3].

The hearing impairment in these patients is quite variable. Deafness ranging from mild to profound can be found. The audiometric threshold pattern can involve only high frequencies or all frequencies. Moreover, deafness may more frequently be cochlear but may also be retrocochlear [4]. 

Magnetic resonance imaging (MRI) represents a fundamental tool for the early diagnosis of MDs, including both conventional MRI imaging and functional imaging (spectroscopy, MRS; diffusion, DWI-ADC; perfusion, DSCI-ASL). Mitochondrial disorders exhibit a wide range of MRI findings, varying according to the clinical phenotype, stage of the disease, and patient age. Even if no single feature is diagnostic, three main MR patterns may be found. In the non-syndromic diseases of adult-hood, the MR pattern is commonly non-specific (pattern type I), characterized by a variable degree of brain atrophy and multifocal cerebral white matter involvement. Typical imaging findings may be found in patients with syndromic phenotypes, either as a specific pattern (type II) or as a leukodystrophy-like pattern (type III). The predominant findings of pattern type II include basal ganglia and brainstem nuclei involvement, along with cortical infarct-like involvement. Atrophy of a variable degree may also be present. Lastly, pattern type III shows the diffuse and symmetric involvement of cerebral deep white matter, progressing toward the subcortical regions. White matter rarefaction with the appearance of small cysts, along with ventricular enlargement, has also been documented within the affected areas. Some cases may also display infratentorial white matter involvement, either as signal alterations in brain-stem long tracts or in cerebellar white matter. In pattern type III, basal and brain stem nuclei are rarely affected [5].

Maternally inherited diabetes and deafness (MIDD) is characterized by maternally transmitted diabetes and sensorineural deafness, while MELAS syndrome is a multi-organ disease with broad manifestations, including stroke-like episodes, dementia, epilepsy, lactic acidemia, myopathy, recurrent headaches, hearing impairment, diabetes, and short stature (Table 1). Hearing loss and diabetes are the most frequent clinical features of MELAS, followed by stroke-like episodes. Patients affected by MIDD may have less severe disease and are susceptible to multisystem complications with heart involvement and stroke-like episodes (Table 1) [1]. Both MELAS and MIDD are frequently related to the m.3243A > G mutation in the mt-tRNA leucine gene (MTTL-1), one of the most common mtDNA point mutations, with a prevalence of 1/400 in the population [6,7]. Interestingly, isolated sensorineural hearing loss occurred associated with m.3243A > G mutation in 3% [7].

CPEO phenotypes of are Kearns–Sayre syndrome (KSS), Pearson syndrome, and progressive external ophthalmoplegia (CPEO). The following triad defines KSS: onset before the age of 20, CPEO, and pigmentary retinopathy (Table 1). Pearson syndrome is characterized by refractory sideroblastic anemia during infancy, and CPEO is marked by progressive bilateral ptosis and a diffuse reduction in ocular motility (Table 1). Another related rare disease is Leigh syndrome (Table 1) [8]. This syndrome (also named subacute necrotizing encephalomyelopathy) is characterized by an onset between 3 and 12 months of age, and its clinical features include neurological signs (hypotonia, spasticity, movement disorders, cerebellar ataxia, and peripheral neuropathy) and extraneurological manifestations (cardiological and respiratory insufficiency). CPEO has mainly been related to single or multiple mtDNA deletions [9]. However, sporadic autosomal dominant or recessive traits involving nuclear genes related to mtDNA or mitochondrial adenine nucleotide replication have been described [10]. Hearing loss was reported in 55, 3% of CPEO patients and in 80% of KSS patients [9]. 

Finally, MERRF is a rare mitochondrial disease with an early childhood or adult onset. It is a multisystem disorder characterized by myoclonus, generalized epilepsy, ataxia, weakness, exercise intolerance, and dementia. The most frequent clinical signs are ptosis, hearing loss, short stature, optic atrophy, cardiomyopathy, cardiac arrhythmias, peripheral neuropathy, pigmentary retinopathy, optic neuropathy, diabetes mellitus, and lipomatosis (Table 1). MERRF is mainly related to the 8344A.G mitochondrial DNA mutation. The prevalence of hearing loss in cohort studies ranges from 40% to 72% (respectively, in cohorts of Italian and German patients), thus resulting in one of the most frequent symptoms [11,12].

The aim of this article was to describe a cross-sectional cohort study of adult patients with genetically defined mitochondrial diseases based on the prevalence and characterization of hearing impairment and neuroimaging results.

## 2. Materials and Methods

This was a retrospective observational study.

### 2.1. Participants

Thirty-four adult patients (mean age: 50.02 ± 15 years, range: 18–75 years; 20 females and 14 males) were recruited over a period of 17 months at the Neurological and ENT Clinics of the Fondazione Policlinico Universitario A. Gemelli, IRCCS. 

All subjects participated voluntarily and gave informed consent (as required by hospital protocols). 

We reviewed the clinical records of patients affected by specific mitochondrial diseases diagnosed using muscle biopsies performed during the initial investigations.

The inclusion criterion was a proven mtDNA or nuclear DNA associated with an MD mutation. 

The exclusion criteria were known otological diseases or exposure to noise and other systemic and neurological diseases that can modify hearing function.

All patients underwent a comprehensive audiological evaluation. We considered a single assessment of the auditory function. 

Patients were classified according to clinical features into four groups: MELAS, MIDD, PEO, and Encephalopathy/Polyneuropathy.

### 2.2. Audiological Evaluation

The audiological evaluations included psychoacoustical tests (pure-tone audiometry and speech audiometry), electrophysiological tests (Auditory Brainstem Responses (ABRs)), and Impedenzometry (tympanometry and Acoustic Reflex Threshold (ART)). An experienced ENT doctor evaluated and interpreted the auditory function according to clinical standards. 

Pure-tone air conduction and bone conduction hearing tests were administered to all participants following standard procedures (Guidelines for Manual Pure-Tone Threshold Audiometry, 2005). The tests were performed in a sound-treated booth with environmental noise of less than 20 dB in order to detect the auditory threshold (Pure-Tone Average (PTA)) of the hearing level in decibels (dBHL). Evaluations of air conduction were obtained using headphones (Telephonics TDh4) for frequencies of 125, 250, 500, 1000, 2000, 4000, and 8000 Hz. Bone conduction was evaluated using a bone conductor for frequencies of 250, 500, 1000, 2000, and 4000 Hz. Hearing loss (HL) was present if the average threshold was a >20 dB hearing level (HL).

The air conduction threshold, bone conduction threshold, and bone–air conduction difference were used to differentiate hearing loss: conductive hearing loss (normal bone conduction threshold and an air conduction threshold of  ≥25 dB HL); sensorineural hearing loss (SNHL) (abnormal threshold of bone conduction and air conduction with a difference of  ≤10 dB HL), and mixed hearing loss (bone conduction difference of  >10 dB HL). The severity of hearing loss was classified as follows: average thresholds ranging between 21 and 40 dB HL were considered mild hearing loss, thresholds of 41–70 dB HL were considered moderate, thresholds of 71–95 dB HL were considered severe, and thresholds >95 dB HL were considered profound. 

Speech audiometry was obtained using monosyllabic words, and the total number of correctly recognized words (as a percentage) was determined for different presentation levels (in dB HL). 

ABRs were recorded using a clinical audiological evoked potential recording device in order to investigate retrocochlear auditory neural processing up to the inferior colliculus. The EEG signal was amplified (100 K) and filtered with a bandpass between 10 and 3000 Hz. The stimulus was an alternating polarity click with a 0.1 ms duration, presented monaurally through earphones (Telephonics TDh4) at the supraliminal level. The total number of stimuli was between 500 and 2000. An analysis of brain stem responses was obtained by evaluating the absolute peak latencies of reproducible waves (I, III, and V), as well as the intervals between the I-III, I-V, and III-V waves. The aim of the ABR test was to define a possible abnormal prolonged neural conductivity between different auditory generators. 

Tympanometry and the ART were assessed using a tympanometric system, inserting a pressurized probe into the patient’s ears. Tympanometry measured the response of the tympanic membrane to changes in pressure, and it was used to study the condition of the middle ear. The ART test was performed by eliciting the contraction of the stapedii muscles with sounds of different frequencies (500, 1000, 2000, and 4000 Hz) with the intensity varying between 65 dB and 95 dB. Both the tympanometry and ART test were helpful in distinguishing between different types of hearing loss, which was useful in the differential diagnosis between cochlear and retrocochlear damage.

### 2.3. Neuroimaging Evaluation

MRI examinations were performed using a 1.5 T MR system (Signa Excite 2 Echospeed, GE Healthcare, Milwaukee, WI, USA) equipped with an eight-channel head coil for sensitivity encoding.

The MRI exams included conventional anatomical sequences such as axial T2-fluid-attenuated inversion recovery (FLAIR), T2 fast spin echo (FSE), axial pre- and post-contrast T1 FSE, post-contrast 3D-fast spoiled gradient echo (FSPGR), susceptibility-weighted imaging (SWI), and diffusion-weighted imaging (DWI). Apparent diffusion coefficient (ADC) maps were automatically calculated from the diffusion data by the scanner software. The following imaging findings were considered: cerebellar atrophy; cerebral cortical and subcortical atrophy; symmetric signal changes in subcortical structures (basal ganglia, brainstem, and cerebellum); asymmetric signal changes in the cerebral cortex, such as stroke-like lesions and laminar necrosis; white matter abnormalities, including leukoencephalopathy; and cysts or vacuolated lesions. These elementary MRI abnormalities could be combined in various ways in a single patient, corresponding to the clinically recognized pathological patterns.

MRI examinations were not performed for four patients for technical reasons (an inability to perform the examination).

The audiological results obtained from the subjective and objective tests were then matched with the genetic data and the neuroradiological findings.

## 3. Results

### 3.1. Genetic Findings

The A-to-G point mutation at position 3243 in the mitochondrial genome (m.3243A > G) was found in 19 patients enrolled in this study (6 with MELAS, 12 with MIDD, and 1 with PEO) (Table 2, Table 3 and Table 4). In total, 11 out of 34 patients showed a single mtDNA deletion (all affected by PEO) (Table 4). Three patients, instead, had alterations of nuclear genes associated with mitochondrial diseases (one PEO patient involving POLG1 and two patients involving OPA1 and suffering from Encephalopathy and Polyneuropathy) (Table 4 and Table 5). Finally, a patient with PEO showed multiple mtDNA deletions without an identified nuclear defect (Table 4).

### 3.2. Audiological Results

Sensorineural hearing loss was present in 25/34 patients. The hearing loss was bilateral and symmetrical and ranged from subclinical to severe (nine mild, nine moderate, five severe, and two profound). The analysis of audiometric pattern showed that 11/25 hearing loss patients had an involvement of all frequencies (flat shape) while in 14/25 patients only high frequencies were involved (ski sloping). Severe or profound hearing loss was always associated with a flat shape type, while mild HL was associated with the ski-sloping one. In the nine patients with moderate hearing loss, we highlighted five ski-sloping curves and five flat shapes (Table 2, Table 3, Table 4 and Table 5). On the contrary, we did not highlight any up-sloping audiometric curves, i.e., involving only the low frequencies.

Considering the electrophysiological data (ABR) and the Acoustic Reflex Threshold (ART), we identified cochlear or retrocochlear origins of deafness: 21 out of 25 patients showed a cochlear site of lesion and four out 25 retrocochlear (Table 2, Table 3, Table 4 and Table 5). All patients with retrocochlear hearing loss showed the ski-sloping audiometric type.

### 3.3. Neuroimaging Results

Neuroradiological evaluations were conducted on 30 patients. We observed global MRI abnormalities or structural brain changes in 26 out of 30 subjects. Twenty-one out of 26 cases exhibited white matter abnormalities, followed by cortical atrophy (15 subjects), subcortical atrophy (10 subjects), basal ganglia involvement (8 subjects), cerebellar atrophy (8 subjects), stroke-like lesions or laminar necrosis (4 subjects), and cysts or vacuolated lesions (1 patient). Stroke-like lesions affecting the parieto-occipital regions and laminar necrosis were characteristic findings of MELAS, predominant cerebral and/or cerebellar atrophy were observed in the PEO group, and white matter changes were core features of the leukoencephalopathy group, whereas nonspecific findings occasionally associated with basal nuclei involvement were observed in the MIDD group (Table 2, Table 3, Table 4 and Table 5).

### 3.4. Audiological and Neurological Results in Different Groups

MELAS group (six patients): The degree of hearing loss ranged from mild (two cases) to severe (only one case). Half of the cases were moderate. Half of the patients showed a flat audiometric pattern, and half showed a ski-sloping pattern. Only one patient had moderate retrocochlear deafness. All patients except one showed cortical atrophy and subcortical atrophy. Four subjects demonstrated stroke-like lesions and laminar necrosis; three had cerebellar atrophy, basal nuclei involvement, and white matter abnormalities; and only one patient had cysts or vacuolated lesions (Table 2).

MIDD group (twelve patients): Three subjects had normal hearing, three had mild ski-sloping hearing loss, four had moderate hearing loss (three of which had the ski-sloping type), and two had profound hearing loss. Three patients had moderate retrocochlear deafness. Four patients did not undergo neuroradiological evaluations (due to technical problems), and only one patient had normal neuroimaging. In all seven remaining cases, we demonstrated the presence of white matter abnormalities. Cortical atrophy, basal nuclei involvement, and cerebellar atrophy were present in three cases (Table 3).

PEO group (fourteen patients): Nine patients showed cochlear deafness ranging from mild to severe (four severe, three mild, and two moderate). Of the two patients with moderate deafness, one showed a ski-sloping pattern and the other showed a flat curve. Three patients had normal neuroimaging evaluations. In the other patients (11 subjects), 9 showed white matter abnormalities, 6 showed cortical atrophy, 4 showed subcortical atrophy, and 2 showed cerebellar atrophy and basal nuclei involvement (Table 4).

Encephalopathy/Polyneuropathy (two patients): One patient affected by Encephalopathy had normal hearing, while the other one had mild cochlear deafness. Both patients showed white matter abnormalities on their MRI (Table 5).

### 3.5. Hearing Loss and Mitochondrial Genotype

In total, 17 patients out of 19 carrying the m.3243A > G mutation had hearing loss ranging from subclinical to severe with predominantly high-frequency involvement; 13 of these presented a cochlear site of lesion, and in the remaining 4 (23.5%) we identified central auditory pathway involvement (Table 2, Table 3 and Table 4).

Eight out of eleven patients with single mtDNA deletion presented a sensorineural hear-ing loss, with cochlear function affected in all cases. Seven patients showed severe hearing loss, while three showed mild hearing loss. Only one patient had moderate deafness associated with a flat curve (Table 4).

Of the patients with nuclear genes associated with mitochondrial diseases (POLG1 and OPA1), only one patient with OPA1 affected by polyneuropathy showed mild cochlear hearing loss (Table 5).

The clinical data are summarized in Table 2, Table 3, Table 4 and Table 5.

## 4. Discussion

Inner ear involvement in MDs has been extensively described in the literature, although the series reported are very often quite small. Our results confirmed that the hearing loss is related to several MDs, represented in all groups with varying degrees and shapes for different sites of injury. 

In particular, sensorineural hearing loss was present in all MELAS patients, and there was retrocochlear involvement in only one patient. Our findings are in partial agreement with the literature, showing that hearing loss is present in 30–81% of patients with MELAS syndrome, varying from mild loss to severe with both cochlear and retrocochlear origins [13,14,15,16]. Like van Kempen [16], we highlighted a downward sloping hearing loss for the highest frequencies, although we also observed the involvement of all frequencies, especially in the most severe hearing losses.

All patients of the MELAS group exhibited different MRI signal abnormalities, in-cluding white matter lesions (both non-cavitating and cavitating leukoencephalopathy), gray matter lesions (involving cortical and subcortical regions), and parenchymal atrophy. However, the distinguishing feature was the occurrence of stroke-like lesions, as reported in the literature [17]. These frequent damages, such as localized areas of increased signal intensity on T2-weighted and FLAIR (fluid-attenuated inversion recovery) sequences, predominantly affect cortical and subcortical regions, specifically in the parieto-occipital lobes. Furthermore, these lesions can sometimes involve the watershed areas. No patient had normal neuroimaging.

Genetically, all subjects affected by MELAS had the DNA (mtDNA) point mutation m.3243A > G, which can therefore be correlated with a more serious disease. The m.3243A > G (A3243G) mutation is a point mutation at base pair number 3243 in mtDNA, resulting in an adenosine to guanine switch in the tRNA-leu (UUR) encoded by the MT-TL1 gene [18]. The phenotypic expression of the m.3243A > G mutation can be extremely variable, ranging from asymptomatic to highly debilitating syndromes, probably depending on the level and distribution of m.3243A > G heteroplasmy across cells and tissues [14,19].

The same genetic observations could be made in the MIDD group, which presented, in our series, the genetic profile of the MELAS group (m.3243A > G mutation) with quite variable clinical manifestations. In this group, we found 2 cases of deafness of a profound degree, 3 of retrocochlear origin, and only 3 of 12 (25%) with normal hearing. Also, in this group we highlighted a greater involvement of the high frequencies, in agreement with the literature [16], with the presence of flat shapes above all in the highest degree of hearing loss. The patients’ age of deafness was more than 41 years, with the exception of one patient who was 28 years old. This finding is in agreement with Tsang et al. [20], who reported the onset of diabetes and deafness during the third or fourth decade of life. 

In the neuroimaging, the MIDD group displayed non specific MRI findings. Specifically, three patients exhibited neuroradiological findings resembling those observed in the MELAS group, while four patients demonstrated only white matter abnormalities with varying configurations, extensions, and distributions. These abnormalities were characterized by increased T2 signals, indicating demyelination, gliosis, or axonal loss [21]. Among the subjects, only one individual with mild cochlear deafness did not exhibit neurological abnormalities. We can hypothesize that the neurological characteristics of the MIDD patients are generally less serious than those of MELAS patients, thus confirming the heteroplasmy influence [22].

In the PEO group, the genetic analysis demonstrated different changes: multiple mtDNA deletions in one subject, POLG1 involvement in another one, and single mtDNA deletions in the remaining subjects. In total, 5 out of 14 patients had normal hearing without any specific genetic pattern. In the PEO group, mild-to-moderate generalized cerebral and/or cerebellar atrophy, which varied in degree, was the only predominant neuroradiological finding. These changes were observed in eight cases and were sometimes accompanied by white matter lesions (WMLs). The presence of these findings represents a challenge in differentiating PEO from other syndromic phenotypes of mitochondrial disorders. Additionally, isolated cases of WMLs were identified in three subjects, while three other subjects exhibited normal MRI findings. Among the three subjects with normal neuroimaging, one had normal hearing. It is noteworthy that all of our patients exhibited a single mutation in the mitochondrial DNA (mtDNA). It can be assumed that the PEO group showed less severe neurological manifestations than the other groups, while four patients demonstrated severe cochlear hearing loss [23]. Our audiological results are in partial agreement with Kornblum et al. [24]: they found a 58.8% rate for hearing loss in PEO patients, while our rate was 64.2%. In addition, they found mild and moderate deafness involving high frequencies, while in our investigation we also demonstrated four patients with severe flat hearing loss.

Therefore, considering our overall results, we can claim that different genetic alterations are associated with different clinical presentations. The genotype–phenotype relationship in mitochondrial disorders is complex, as previously observed: a single mutation can cause several clinical syndromes, while different genetic alterations can cause similar phenotypes [16,25].

The improvement of knowledge could be implemented by large multicenter studies, contributing to better patient categorization, which is useful to understand the natural history of mitochondrial disorders.

A bias of this study is the lack of an applied correction factor for age (as recently performed by van Kempen [16]). However, it should be noted that the age of our patients was low, with only eight patients over the age of 61.Therefore we should not have overestimated hearing loss in specific groups of MDs.

## 5. Conclusions

MDs are pathological conditions with high genotypic and phenotypic variability.

The associated hearing loss is usually bilateral but it can have different qualitative and quantitative characteristics. Deafness can be progressive, cochlear, or retrocochlear, with different shapes. MRI lacks the high sensitivity or specificity for diagnosing the syndromic forms of mitochondrial diseases, although certain disease types may exhibit more distinguishing brain imaging features or patterns. The other important symptoms and signs (neuromuscular, ocular, and endocrine) related to the different syndromic diseases must also be well known in order to define a correct diagnosis. Achieving an accurate diagnosis and the effective management of these conditions requires a comprehensive diagnostic evaluation, which includes clinical assessment, genetic testing, and auditory investigations. Auditory dysfunction should alert physicians requiring confirmation of the diagnosis with genetic testing.

Further studies on the mitochondrial mechanisms involved the inner ear are needed to better understand the true prevalence and expression of the hearing impairment in MDs.

Finally, it will be important to establish the follow-up-times for patients with hearing loss in order to improve the understanding of different audiological phenotypes for both qualitative and quantitative outcomes.

## Figures and Tables

**Table 1:** Clinical characteristics of mitochondrial diseases.

MELAS	stroke-like episodes, dementia, epilepsy, lactic acidosis, myopathy, recurrent headaches, hearing impairment, diabetes, short stature
MIDD	diabetes, deafness, myopathy, congestive heart failure, muscular dystrophy, short stature, constipation, malabsorption, cerebellar and cerebral atrophy
KSS	pigmentary retinopathy, PEO, cerebellar ataxia, impaired intellect (intellectual disability, dementia, or both), sensorineural hearing loss, ptosis, oropharyngeal and esophageal dysfunction, exercise intolerance, muscle weakness, cardiac conduction block, endocrinopathy
Pearson syndrome	life-threatening sideroblastic anemia, exocrine pancreatic dysfunction
PEO with exercise intolerance	ptosis, impaired eye movements due to extraocular muscle paralysis (ophthalmoplegia), oropharyngeal weakness, variably severe proximal limb weakness
Leigh syndrome	bilateral and slowly progressive loss of extraocular muscle mobility, orbicularis oculi weakness, ptosis, other neuromuscular symptoms
MERRF	myopathy, cardiomyopathy, ataxia, epilepsy, lipomatosis, neuropathy, migraine, deafness

**Table 2:** Description of genotypes and phenotypes (audiological and neurological findings) of MELAS patients.

ID	Age	Sex	Mutation	Phenotype	HL	Site of Lesion	Neuroimaging
1	35	M	m.3243A > G	MELAS	Severe (flat shape)	Cochlear	Cortical atrophy, subcortical atrophy, stroke-like lesions, laminar necrosis, basal nuclei involvement, cerebellar atrophy
2	64	F	m.3243A > G	MELAS	Moderate (flat shape)	Cochlear	Cortical atrophy, subcortical atrophy, white matter abnormalities, stroke-like lesions, laminar necrosis, cerebellar atrophy
3	48	F	m.3243A > G	MELAS	Mild (ski-sloping)	Cochlear	Cortical atrophy, subcortical atrophy, white matter abnormalities, stroke-like lesions, laminar necrosis, cerebellar atrophy
4	19	F	m.3243A > G	MELAS	Mild (ski-sloping)	Cochlear	Cortical atrophy, subcortical atrophy, basal nuclei involvement, cysts or vacuolated lesions
5	38	M	m.3243A > G	MELAS	Moderate (ski-sloping)	retrocochlear	Cortical atrophy, subcortical atrophy, stroke-like lesions, laminar necrosis, basal nuclei involvement
6	47	M	m.3243A > G	MELAS	Moderate (flat shape)	cochlear	White matter abnormalities

**Table 3:** Description of genotypes and phenotypes (audiological and neurological findings) of MIDD patients.

ID	Age	Sex	Mutation	Phenotype	HL	Site of Lesion	Neuroimaging
1	53	F	m.3243A > G	MIDD	Mild (ski-sloping)	cochlear	White matter abnormalities
2	53	F	m.3243A > G	MIDD	Moderate (ski-sloping)	retrocochlear	White matter abnormalities
3	51	F	m.3243A > G	MIDD	Moderate (ski-sloping)	retrocochlear	Cortical atrophy, white matter abnormalities, basal nuclei involvement, cerebellar atrophy
4	29	M	m.3243A > G	MIDD	Normal	-	Not performed
5	34	M	m.3243A > G	MIDD	Normal	-	Not performed
6	28	M	m.3243A > G	MIDD	Mild (ski-sloping)	cochlear	Normal
7	56	M	m.3243A > G	MIDD	Profound (flat shape)	cochlear	Not performed
8	53	M	m.3243A > G	MIDD	Moderate (flat shape)	cochlear	Cortical atrophy, subcortical atrophy, white matter abnormalities, basal nuclei involvement, cerebellar atrophy
9	59	F	m.3243A > G	MIDD	Mild (ski-sloping)	cochlear	Not performed
10	61	F	m.3243A > G	MIDD	Profound (flat shape)	cochlear	Cortical atrophy, white matter abnormalities, basal nuclei involvement, cerebellar atrophy
11	18	M	m.3243A > G	MIDD	Normal	-	White matter abnormalities
12	41	F	m.3243A > G	MIDD	Moderate (ski-sloping)	retrocochlear	White matter abnormalities

**Table 4:** Description of genotypes and phenotypes (audiological and neurological findings) of PEO patients.

ID	Age	Sex	Mutation	Phenotype	HL	Site of Lesion	Neuroimaging
1	75	F	m.3243A > G	PEO	Moderate (ski-sloping)	cochlear	Cortical atrophy, white matter abnormalities, cerebellar atrophy
2	51	F	multiple mtDNA deletions	PEO	Normal	-	Cerebellar atrophy
3	65	M	POLG1	PEO	Normal	-	Cortical atrophy
4	74	F	single mtDNA deletion	PEO	Severe (flat shape)	cochlear	Cortical atrophy, white matter abnormalities
5	39	F	single mtDNA deletion	PEO	Normal	-	Normal
6	52	F	single mtDNA deletion	PEO	Normal	-	White matter abnormalities
7	61	F	single mtDNA deletion	PEO	Mild (ski-sloping)	cochlear	Normal
8	65	M	single mtDNA deletion	PEO	Moderate (flat shape)	cochlear	Cortical atrophy, subcortical atrophy, white matter abnormalities
9	31	M	single mtDNA deletion	PEO	Mild (ski-sloping)	cochlear	Cortical atrophy, subcortical atrophy, white matter abnormalities
10	70	F	single mtDNA deletion	PEO	Mild (ski-sloping)	cochlear	Normal
11	69	F	single mtDNA deletion	PEO	Severe (flat shape)	cochlear	Cortical atrophy, subcortical atrophy, white matter abnormalities
12	66	M	single mtDNA deletion	PEO	Severe (flat shape)	cochlear	Cortical atrophy, subcortical atrophy, white matter abnormalities, basal nuclei involvement
13	49	F	single mtDNA deletion	PEO	Severe (flat shape)	cochlear	White matter abnormalities
14	47	F	single mtDNA deletion	PEO	Normal	-	White matter abnormalities, basal nuclei involvement

**Table 5:** Description of genotypes and phenotypes (audiological and neurological findings) of patients affected by Encephalopathy and Polyneuropathy.

ID	Age	Sex	Mutation	Phenotype	HL	Site of Lesion	Neuroimaging
1	43	F	OPA1	Encephalopathy	Normal	-	White matter abnormalities
2	57	M	OPA1	Polyneuropathy	Mild (ski-sloping)	cochlear	White matter abnormalities
